# A machine learning web application for screening social anxiety disorder based on participants’ emotion regulation (ML-SAD)

**DOI:** 10.3389/frobt.2025.1620609

**Published:** 2025-09-25

**Authors:** Sara Ahmadi Majd, Mohamad Rasoul Parsaeian, Mohsen Madani, Hadi Moradi, Abolfazl Mohammadi

**Affiliations:** 1 Campus Institute Data Science, Georg-August-Universität Göttingen, Göttingen, Germany; 2 College of Psychology and Educational Sciences, University of Tehran, Tehran, Iran; 3 College of Electrical and Computer Engineering, University of Tehran, Tehran, Iran; 4 Department of Psychiatry, Tehran University of Medical Sciences, Tehran, Iran

**Keywords:** social anxiety disorder, emotion regulation, machine learning, web application, screening tools

## Abstract

Social Anxiety Disorder (SAD) is called a neglected anxiety disorder since people do not realize its existence and the need to receive further treatment. Thus, it is essential to develop widely available self-screening systems to assess individuals and direct those who need further evaluation to appropriate resources. Consequently, this paper presents a web application based on machine learning to screen for SAD. The Web application comprises 10 multimedia scenarios that people with SAD may struggle with. Four hundred and eighty-eight young adults (18–35 years old) in Persian-speaking society were asked to consider themselves in these scenarios and rank their competency in dealing with each specific situation, considering three emotion regulation strategies. Participants were divided into two groups, SAD and non-SAD, based on their diagnostic history of SAD and their self-assessment of their anxiety level. Multiple machine learning models were trained and evaluated, achieving an accuracy rate of more than 80% and demonstrating the effectiveness of the tool in identifying individuals who need additional support.

## 1 Introduction

Social Anxiety Disorder (SAD) is a persistent and intense fear of a social situation in which the individual believes that they may be humiliated, embarrassed, or negatively judged (Association and Association, 2013). SAD may disrupt all aspects of a person’s life, with problems in education, work, and personal relationships. For example, people with SAD have been shown to have higher unemployment and reduced marriage rates compared to normal people (Wittchen et al., 2000). The high prevalence of this disorder, that is, about 12% of the population, has led it to be fifth among psychiatric disorders (Alonso et al., 2004). Unfortunately, like most psychiatric disorders, the diagnosis methods of SAD are based on interviews and clinical assessments (Nordgaard et al., 2012). Consequently, this results in the limited availability of these methods, especially in rural areas with limited access to experts (Hauenstein et al., 2007). Furthermore, questionnaires are subjective and results may differ according to the responses of interviewees and interviewers.

Furthermore, most people with SAD do not go to an expert to be diagnosed and receive additional treatments. That is why this disorder is often called neglected anxiety disorder (Nagata et al., 2015). As a result, it is likely to be diagnosed when SAD patients visit an expert for other disorders such as mood, OCD, alcohol use, or avoidant personality disorders (Liebowitz, 1999; Koyuncu et al., 2019).

Therefore, a widely available self-screening approach may be beneficial for patients to become aware of their disorder (Romijn et al., 2019). That is why in this paper, we present a web-based SAD screening system that can be accessed on various electronic devices. In our Web-based system, instead of basic questions in standard questionnaires, ten multimedia scenarios were developed to measure the self-regulation capabilities of people. In other words, we attempted to simulate an evaluation session conducted by an expert in which an examiner inquires about a subject’s feelings in various situations. In addition, a machine learning approach was employed to train a system that automatically screens users and provides recommendations for subsequent actions.

Although our scenario-based machine learning web application enhances reach and objectivity, it still relies on users’ willingness to engage with a screen. Recent advances in socially assistive robotics suggest a complementary approach: Embodied agents that can deliver the same standardized assessment in a more naturalistic, face-to-face manner, potentially reducing avoidance and enhancing trust for people with high social anxiety. Therefore, the following subsection considers how social assistive robots can extend and amplify the benefits of our screening application.

### 1.1 Social assistive robots for mental health screening and support

Socially assistive robots (SARs) are embodied agents that provide help through social rather than physical interaction, offering scalable and continuously available support in the healthcare domains (Feil-Seifer and Matarić, 2005). Because SARs can present standardized prompts while capturing rich behavioral signals (gaze, posture, prosody), they are increasingly being explored as front-line tools for mental health screening (Rasouli et al., 2022).

For individuals with SAD, the first interaction with a non-judgmental robot can reduce fears of negative evaluation and increase willingness to disclose sensitive information (Rasouli et al., 2022). Empirical work shows that SARs that exhibit warm non-verbal behaviors such as open arm gestures, adaptive gaze, or slow “breathing” motions can reduce state anxiety, foster trust, and support emotion regulation (Williams et al., 2023; Klausen et al., 2025; Webb et al., 2024). Coupling such expressive agents with our scenario-based web application could therefore further lower access barriers and create a more engaging assessment environment for people who might otherwise avoid traditional evaluations.

### 1.2 Related works

The SAD assessment process involves the use of questionnaires such as the Liebowitz Social Anxiety Scale (Liebowitz, 1987) and the Social Phobia Inventory (Connor et al., 2000). The Liebowitz Social Anxiety Scale is a 24-item questionnaire that measures the rate of fear and avoidance in different social situations. People must rate how much fear they experience (none, mild, moderate, or severe) and how often they have avoidance behavior (never, occasionally, frequently, or usually) (Liebowitz, 1987).

The Social Phobia Inventory (SPIN) is a 17-item questionnaire to screen and measure the severity of SAD. This questionnaire has five main factors related to 1) fear and avoidance of talking to strangers or social gatherings, 2) self-esteem and fear of criticism, 3) physiological symptoms, 4) social inferiority with fear and avoidance of authority, and 5) avoidance of attention to oneself, specifically being a center of attention. Unfortunately, these questionnaires are primarily administered and interpreted by experts, which limits their widespread availability. Furthermore, experts’ knowledge may influence the quality of assessments. To address these issues, there are various studies on the use of machine learning to detect cognitive deficits such as SAD and depression. For example, a study used machine learning to demonstrate a novel approach to classifying depression, rather than conventional norm-based methods (Yang et al., 2020). Another study illustrated the promise of machine learning technology in predicting anxiety (Sau and Bhakta, 2017). For the prediction of SAD, one study demonstrated that the presence or absence of SAD, as well as the level of response to treatment, could be classified using machine learning algorithms. Muhammad et al. (2020). Therefore, we used machine learning approaches to screen for SAD.

However, interviewees may not clearly understand the situation, and their answers may be biased in face-to-face interviews (Nordgaard et al., 2013). Consequently, our system uses multimedia scenarios to engage users and help them better understand the situations discussed, providing reliable answers. For the prediction of SAD, one study demonstrated that the presence or absence of SAD, as well as the response levels of treatment, could be classified using machine learning algorithms (Muhammad et al., 2020). Therefore, we found it helpful to use machine learning in our study.

Our scenarios are based on many studies that show that SAD is associated with emotional dysregulation. People with SAD have difficulty regulating their emotions (Hofmann, 2007); But so far, the problem of emotion regulation has not been introduced as a diagnostic criterion in (Association and Association, 2013). There has also been a reported mismatch between existing diagnostic criteria for SAD and research (Jazaieri et al., 2019).

According to the Gross model, emotion regulation refers to conscious or unconscious processes by which individuals influence their emotions (Gross, 1998). Emotion regulation can affect different aspects of emotion, such as the type, intensity, frequency, and duration of emotion (Gross and Jazaieri, 2014).

Studies have shown that emotion regulation strategies are divided into five groups: situation selection, situation modification, attentional deployment, cognitive change, and response regulation (Gross, 1998).

Reappraisal is a popular cognitive change model, known as an adaptive emotion regulation strategy, that can modulate an individual’s emotional responses in the face of a stressful situation (Gross and Thompson, 2007). Suppression is a model of response regulation and a maladaptive strategy that prevents emotional responses (Werner et al., 2011). Avoidance is a model of situation selection and a maladaptive strategy in which the individual escapes the situation (Jazaieri et al., 2015).

Studies have shown that healthy individuals are more likely to use reappraisal compared to patients with SAD (Dalton et al., 2025). On the other hand, SAD patients are more likely to use suppression and avoidance strategies compared to healthy individuals (Jazaieri et al., 2015). So, there is a link between SAD and the use of suppression and avoidance, and the absence of the disorder and reappraisal. Thus, we considered these strategies as our feature in screening patients with SAD from those without SAD.

In other words, this paper introduces a new approach to the screening of SAD based on emotion regulation strategies, including avoidance, suppression, and reappraisal. We only considered these three emotion regulation strategies for simplicity. We developed ten multimedia scenarios that simulate the difficulties faced by people with SAD. People with SAD and healthy controls rated the extent to which they used each strategy in each scenario. In addition, we used the data extracted from the ratings to train machine learning algorithms for future screening purposes. In general, this study aims to determine whether emotion regulation can predict SAD using a scenario-based web application and a machine learning approach.

## 2 Methods

### 2.1 Participants

To collect participants, a flyer was prepared that contained an explanation of SAD and a request for cooperation. It was published along with a link to our website on various social media platforms, including Instagram, Telegram, and LinkedIn, in Iranian society. We successfully recruited a total of 495 participants for the study. There were 298 women and 197 men. The average age of the participants was 23 years (M = 23.12, SD = 4.17), with the majority between 18 and 35 years. It appears that since it was offered online, the new generation of Internet-savvy individuals was reached more effectively than other generations (Bardeen and Fergus, 2014; Heeren and McNally, 2016). This age distribution was suitable for the study, as the designed scenarios aligned with it. Therefore, data above or below this age range were deleted.

After this data filtering phase, 488 participants, comprising 294 women and 194 men, remained in the study. The average age of the remaining is 23 years (M = 23.04, SD = 3.97). Of this group of participants, 60 women and 38 men self-reported that they had received an expert diagnosis of SAD (for further reliability of their claim, they were asked to write how they were diagnosed with SAD). Among them, 234 men and 154 women were not diagnosed. Among diagnosed people, 62 of them had “very high” and “high” anxiety levels, 33 had “moderate” anxiety levels, and 3 had “low” and “very low” anxiety levels. Furthermore, 209 of the undiagnosed individuals had “very high” and “high” anxiety levels, 139 had “moderate” anxiety levels, and 42 had “very low” and “low” anxiety levels (Table 1).

**TABLE 1:** Number of people with their diagnostic and anxiety level information.

Anxiety level	Diagnosed?	Number of people
high/very high	yes	62
moderate	yes	33
low/very low	yes	3
high/very high	no	209
moderate	no	139
low/very low	no	42

In this study, all relevant ethical standards were considered. All participants received and signed a consent form, based on the Declaration of Helsinki, which informed them about the study objectives, experimental procedures, and their rights as participants. In addition, there were no reimbursements for participating in the task, and they did so voluntarily.

### 2.2 Materials and procedures

#### 2.2.1 Web application

jQuery and Flask technologies were used to design the frontend and backend, according to HTML5 standards. The link to the tool’s web interface was given to the participants. The scenario for each challenge was based on a social situation in which people with SAD have difficulty dealing with. Previous studies have discussed these scenarios (Gross and John, 2003; Liebowitz, 1987), and an expert also verified these scenarios. The strategy corresponding to each scenario was designed based on emotion regulation strategies, including suppression, avoidance, and reappraisal.

It should be noted that we drew inspiration from the Emotion Regulation Questionnaire (Gross and John, 2003) to design suppression and reappraisal strategies. The validity and reliability of this questionnaire have been assessed and it has been subsequently translated into Persian, followed by validation in the Iranian society (Kazemi et al., 2023). We designed avoidance strategies based on the avoidance subscale of the Liebowitz questionnaire (Liebowitz, 1987), which has also been translated into Persian and validated in Iranian society (Hasani et al., 2017). We conducted the pilot study in collaboration with the university’s mental health clinic (https://counseling.ut.ac.ir/), which allowed us to collect a modest data set. However, because we made several updates to the software and scenarios during the pilot study, these preliminary data were excluded from the final analysis.

We designed a total of 10 scenarios. The participants could rate the concordance between their usual behavior and the responses suggested for each strategy. The rating indicated the degree of concordance with each strategy on a 7-point Likert scale, ranging from 0 “not at all”) to 7 (“very much”). Images of each scenario were designed and sketched to better immerse the participants in the desired situations. A narrator (a female voice for women and a male voice for men) reads each scenario aloud, accompanied by the corresponding image. The narration soundtrack was mixed with the related ambient sound, including noise, door openings, and cell phone ringtones, to make the scenarios more realistic. The scenarios are shown in Table 2.

**TABLE 2:** Title of the social scenarios.

Scenario	Description
1	Mobile ringing in the meeting
2	Talking about yourself at a party
3	Hearing others talk about yourself
4	Criticize your colleagues’ opinion in the meeting
5	Late arrival to the meeting
6	Talking about your interests in public
7	Trying to communicate with a loved one
8	Being in the center of attention and admired by others
9	Honoring others
10	Asking about someone’s personal life

Individuals could access the web page at any time. A brief description of the research, the emotion regulation topic, and SAD was available on the page. Before starting the process, participants read about the ethics of the study, which had been approved by an ethics board. Then, they could proceed further after accepting the invitation to enter the study by signing the consent form. Then they entered a page that contained instructions about the research procedure. They should have entered their email address, age, sex, self-assessment of their anxiety level (very low, low, medium, high, very high), and whether they were diagnosed with SAD. If you have been diagnosed with SAD, they could add the assessment method and/or the name of the specialist who performed the diagnosis. The set of participants who were diagnosed with SAD was used as our ground truth.

**FIGURE 1:**
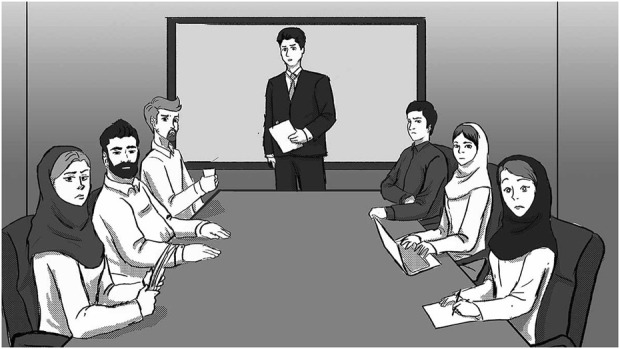
An Example Scenario: Mobile Ringing in the Meeting. I’m in a meeting where my cell phone suddenly starts ringing. I will cut it off immediately. People notice this and look at me.

**FIGURE 2:**
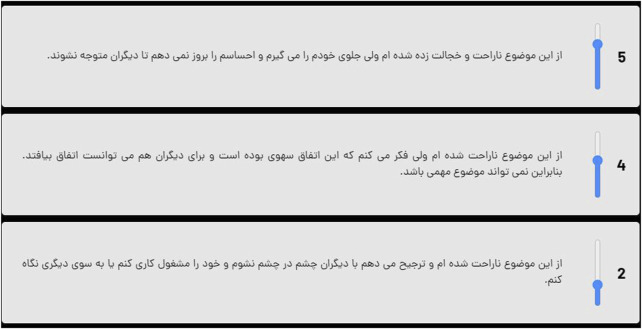
1- I am upset and embarrassed about this. However, I control myself and do not express my feelings, so that others do not notice them (Suppression). 2- I am sorry about this. However, I believe it was an inadvertent mistake that could have occurred in similar situations. Therefore, it cannot be an important case (Reappraisal). 3- I am upset about this and prefer not to face others. Thus, I focus on something else or look at it another way (Avoidance).

The ten scenarios were placed on a page and could be selected using their corresponding buttons. The participants entered each scenario by clicking on the corresponding buttons. The narrators described the scenario while ambient sound was played in the background. After that, the three emotion regulation strategies were displayed on the screen. Participants were asked to score each strategy on a scale of 0–7, using the Likert scale. An example is shown in Figures 1, 2.

On the last page, participants were thanked for their participation in the investigation and asked to click if they wished to receive their results in the future. After the experiment period had finished and the results were ready, we sent the predicted result of the system to those who requested it.

#### 2.2.2 Social phobia inventory (SPIN)

For the validation of the results, a link containing the SPIN questionnaire was sent to the participants through their email addresses. As mentioned in the Introduction, the questionnaire includes 17 questions that participants should respond to regarding their level of agreement with the statements. The scores are “Not at all” (0), “Little” (1), “To some extent” (2), “Much” (3), or “Very much” (4). Finally, the scores are added together. Any score between 0 and 22 is associated with a lack or low SAD, summed scores between 23 and 45 show moderate SAD, and summed scores above 45 suggest high SAD (Connor et al., 2000).

## 3 Result

**FIGURE 3:**
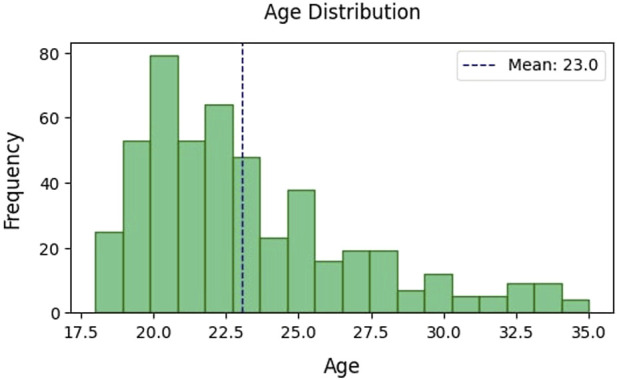
The Age of the participants.

The total number of participants in the 18 to 35-year-old age range was 488. Among them, the number of women was 294, and the number of men was 194. The average age was 23 years (Figure 3).

### 3.1 Statistical results

To evaluate the reliability of our online Web Application, we used the Cronbach alpha coefficient (Lakens, 2022). A Cronbach’s alpha coefficient of 90 or more implies outstanding internal consistency; 80 or better is good, 70 or higher is acceptable, and less than that is poor and in doubt.

We calculated Cronbach’s alpha coefficient for the questions related to suppression, avoidance, and reappraisal strategies. The coefficients for the suppression, avoidance, and reappraisal strategies were 76%, 78%, and 75%, respectively. In other words, these three elements have acceptable reliability.

Furthermore, we focused on two extreme groups along the spectrum of SAD. SAD group: Participants diagnosed with SAD with high (4) or very high (5) anxiety levels reported. No SAD group: Undiagnosed participants with very low (1) or low (2) anxiety levels reported.

We limited our analysis to these extremes for two reasons. First, data was collected online, so we were unable to directly verify self-reported diagnoses; therefore, we required strong claims regarding SAD. Second, participants already diagnosed with SAD may have been undergoing treatment, which could reduce their anxiety in social situations. After applying these criteria, the SAD group consisted of 62 participants and the SAD non-SAD group consisted of 42 participants (Table 3).

**TABLE 3:** Participants are divided into two groups (People with moderate stress level are not considered).

Anxiety level	Diagnosed?	Group	Number of people
high/very high	yes	SAD	62
low/very low	no	No SAD	42

**FIGURE 4:**
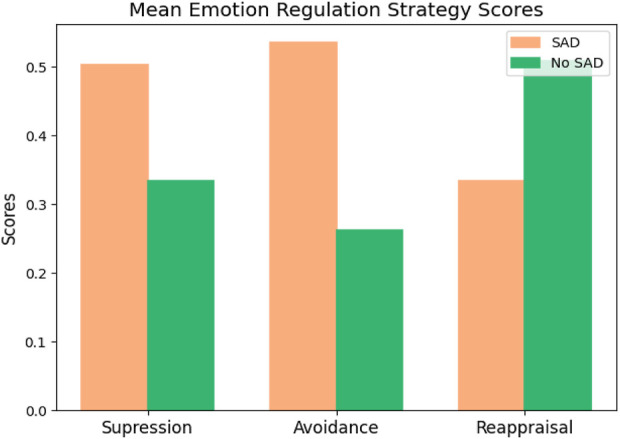
Differences between the use of suppression, avoidance, and reappraisal strategies in two groups: SAD and No SAD. All scores are significant between the SAD and No SAD groups (p-value < 0.001).

**FIGURE 5:**
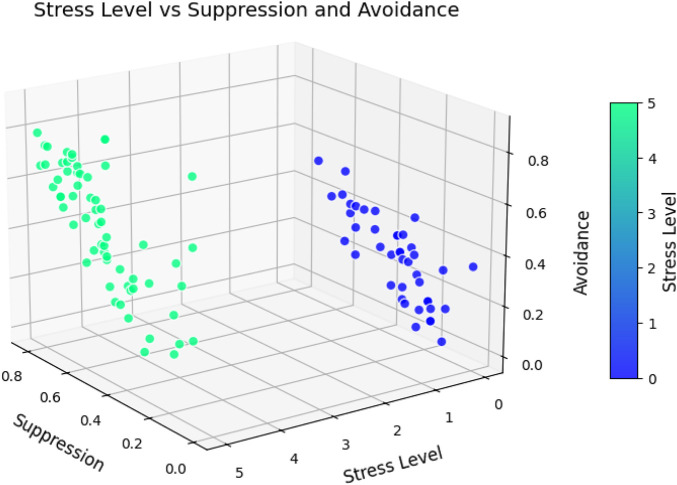
Relationship between stress level and maladaptive emotion regulation strategies: Suppression and Avoidance, among SAD and No SAD groups (People with moderate stress level are not considered). The is a high correlation between these two maladaptive strategies (r = 0.62, p-value < 0.001).

Since the data distributions were not normal, as indicated by the Shapiro-Wilk normality test, we employed the Mann-Whitney test. The results showed a significant difference between the scores in the two groups across all three strategies: Reappraisal, Suppression, and Avoidance (corrected p-value < 0.001). In addition, effect sizes were also implemented (Lakens, 2022), which showed a significant difference in using the strategy between the SAD and non-SAD groups (Table 4; Figures 4, 5.)

**TABLE 4:** Comparisons of emotion regulation strategies between the SAD and No SAD groups (Mann–Whitney U test results after Holm–Bonferroni correction).

Strategy	P-value (holm-bonferroni)	Effect size (rank-biserial)	Interpretation
Reappraisal	< 0.001	−0.56	Sig. lower in SAD group
Suppression	< 0.001	0.48	Sig. higher in SAD group
Avoidance	< 0.001	0.73	Sig. higher in the SAD group

**FIGURE 6:**
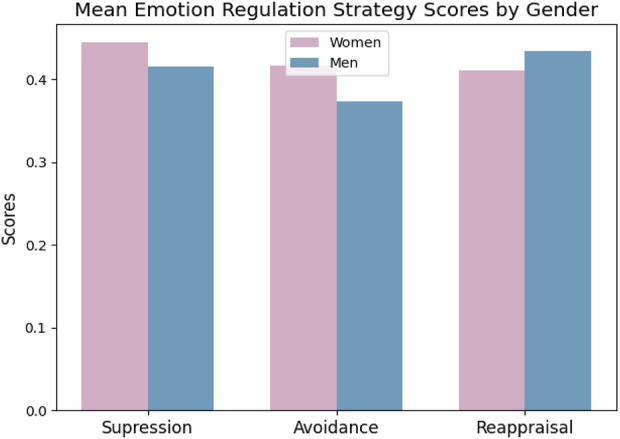
The differences in the use of suppression, avoidance, and reappraisal strategies among women and men (The difference is only significant in using the Avoidance strategy with p-value = 0.015).

The Shapiro-Wilk normality test also did not show normal distributions for reappraisal and suppression strategies among men and women. Thus, the Mann-Whitney test was used. According to the test, there is a significant difference in the use of avoidance strategies between men and women (p-value = 0.014). However, the p-values for the suppression and reappraisal strategies were 0.107 and 0.175, respectively. Therefore, it cannot be argued that men and women applied the strategies completely differently (Figure 6). No significant differences were found between the different age groups in the use of the strategies.

**FIGURE 7:**
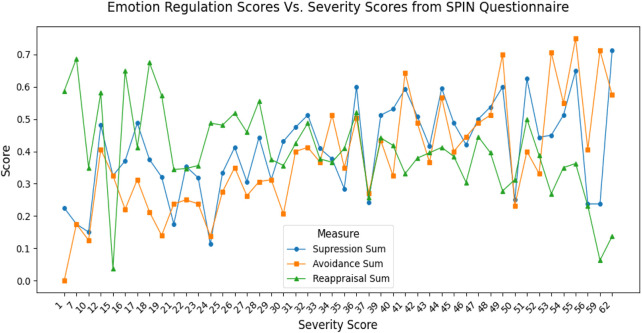
130 people participated in both the ML-SAD web application and SPIN questionnaire. The plot shows an increasing rate of using avoidance and suppression strategies with respect to the Severity Score of the SPIN questionnaire, and a decreasing rate of reappraisal.

Since data collection was conducted online, to improve the validity of our methodological approach, we further analyzed the correlations between the suppression, avoidance, and reappraisal Strategies ratings in the web application and the summed scores of the SPIN questionnaire. Thus, data from the 130 participants who completed both the ML-SAD Web Application and the SPIN questionnaire were used. The correlation between suppression and the questionnaire score was 0.37, and for avoidance and reappraisal was 0.57 and −0.43, respectively (Figure 7). By merging ML-SAD data with SPIN results, the correlation between predefined SAD/no SAD groups and the summed scores of the SPIN questionnaire was 0.77 (33 people).

These two correlation analysis showed two significant points. First, the design and structure of the strategies align with the result of a previously validated questionnaire for SAD. Second, the self-reported approach with the extreme values considered was successful in separating participants with SAD and those without SAD.

We also calculated the correlation between the three strategies. The correlation between suppression and avoidance scores was 0.62, the correlation between avoidance and reappraisal scores was −0.26, and the correlation between suppression and reappraisal scores was 0.04. These findings also demonstrate a strong link between the scores on avoidance and suppression strategies.

### 3.2 Machine learning results

To train the machine learning models, we used 30 individual features representing responses to the three emotion regulation strategies across 10 multimedia scenarios. Demographic variables (e.g., age, sex) were excluded from the analysis. Since each feature (i.e., each strategy within each scenario) demonstrated varying predictive performance (see Tables 5, 6), we retained all features individually rather than aggregating them across scenarios.

**TABLE 5:** Correlation between each strategy score across scenarios and the corresponding total (summed) strategy score.

Scenarios	Suppression	p-val	Avoidance	p-val	Reappraisal	p-val
1	0.61	3.78e-50	0.62	2.24e-52	0.60	2.13e-49
2	0.65	9.81e-61	0.66	2.79e-61	0.52	7.40e-36
3	0.46	2.13e-26	0.54	1.24e-38	0.53	6.22e-37
4	0.59	5.88e-47	0.57	1.87e-43	0.54	1.60e-37
5	0.55	1.04e-39	0.63	1.89e-55	0.62	6.33e-53
6	0.50	2.59e-32	0.60	5.47e-50	0.60	8.23e-49
7	0.47	3.04e-28	0.59	2.95e-46	0.60	3.76e-49
8	0.57	8.78e-43	0.55	2.32e-40	0.55	3.50e-40
9	0.61	6.50e-51	0.63	2.11e-55	0.47	3.06e-28
10	0.57	1.48e-43	0.47	4.00e-28	0.45	1.13e-25

**TABLE 6:** Factor Analysis: Factor loadings for each strategy and scenario. The results indicate that Factor 1 is primarily associated with Avoidance and Suppression, suggesting a link to SAD, while Factor 2 is more strongly associated with Reappraisal, indicating a pattern consistent with No SAD.

Scenarios	Factor 1: SAD			Factor 2: No SAD		
	Avoidance	Reappraisal	Suppression	Avoidance	Reappraisal	Suppression
1	0.50	−0.17	0.52	−0.17	0.50	0.06
2	0.49	0.08	0.60	−0.25	0.48	0.07
3	0.46	0.02	0.32	−0.00	0.43	0.25
4	0.53	−0.10	0.53	−0.22	0.49	−0.05
5	0.55	−0.11	0.53	−0.16	0.55	0.07
6	0.44	−0.08	0.35	−0.33	0.57	0.11
7	0.53	−0.09	0.43	−0.09	0.50	−0.10
8	0.47	−0.06	0.52	−0.07	0.48	0.06
9	0.50	0.02	0.52	−0.22	0.40	0.09
10	0.32	0.17	0.44	−0.14	0.38	0.17

The dataset was split into training (80%) and test (20%) sets. A preprocessing pipeline was applied to handle missing values (using median imputation) and to scale the features via standardization (zero mean and unit variance). This preprocessing was integrated within each fold of the cross-validation pipeline to prevent data leakage and ensure a fair comparison across models.

We employed classification algorithms from the Scikit-learn package (Pedregosa et al., 2018), including Random Forest, Support Vector Machine (RBF kernel), K-Nearest Neighbors and Perceptron. To ensure fairness, all models were subjected to hyperparameter tuning using GridSearchCV with repeated 5-fold stratified cross-validation. Tuning was optimized on F1 score, and the best models were then re-evaluated on accuracy, precision, recall, F1, and ROC AUC. The results are summarized in Table 7.

**TABLE 7:** Accuracy Precision, Recall, F1-score, and ROC AUC of each algorithm after hyperparameter tuning with repeated 5-fold stratified cross-validation.

Algorithm	Accuracy	Precision	Recall	F1	ROC AUC
KNN	**0.84**	0.80	**0.81**	**0.79**	0.88
RF	0.83	**0.88**	0.74	0.77	**0.90**
SVM	0.80	0.80	0.71	0.73	**0.90**
Perceptron	0.76	0.76	0.70	0.704	0.84

• Hyperparameter tuning for all models was performed using GridSearchCV with repeated 5-fold stratified cross-validation. The optimization was based on the F1 score to balance precision and recall, which is particularly relevant in the context of clinical screening.

• Best Parameters:

• KNN: n_neighbors = 5, weights = distance, p = 1

• RF: n_estimators = 200, max_depth = 5, max_features = sqrt, min_samples_split = 5

• SVM: C = 1, kernel = rbf, gamma = scale

• Perceptron: alpha = 0.001, penalty = l2, max_iter = 1000, tol = 0.001

The tuned results indicate that no single model was uniformly superior across all metrics. Instead, each classifier demonstrated distinct advantages and drawbacks:

- K-Nearest Neighbors (KNN): Achieved the highest F1 score (0.79), recall (0.81) and accuracy (0.84), which makes it favorable when minimizing missed cases is the priority.
- Random Forest (RF): Attained the highest precision (0.88) and tied with SVM for the best ROC AUC (0.90), highlighting its strength in reducing false positives.
- Support Vector Machine (SVM): Also achieved a ROC AUC of 0.90, showing strong discriminative ability across thresholds, although with slightly lower recall.
- Perceptron: Performed consistently weaker across all metrics compared to the other models.

Taken together, the results show that each algorithm has specific strengths depending on the clinical emphasis: KNN is preferable when sensitivity is prioritized, RF when reducing false positives and interpretability are more important, and SVM when maximizing discrimination across thresholds is desired. Rather than selecting a single “best” model, our findings emphasize that the choice of classifier should depend on the practical trade-offs relevant to the intended application.

To compare the robustness of ML-SAD and SPIN, we calculated the Sensitivity and Specificity matrices. For the SPIN questionnaire with a sample size of 33, the sensitivity and specificity are 0.47 and 0.50, while for the ML-SAD test dataset with a sample size of 32 these are 0.79 and 0.78 (not cross-validated; the results may vary with different train–test splits but remain significantly greater than SPIN, see Table 8). For both sensitivity and specificity, results increase in ML-SAD compared to SPIN, indicating robustness in detecting true cases as well as excluding negative ones.

**TABLE 8:** Comparison between the performance of ML-SAD classifier with the SPIN questionnaire.

Metric	ML-SAD (n_test = 32)	SPIN (n = 33)
Sensitivity	0.79	0.68
Specificity	0.78	0.21

Labeling of SPIN questionnaire data (Connor et al., 2000):

• Total score 0–22 → label 0

• Total score 23–45 → not considered

• Total score 46–68 → label 1

## 4 Discussion

In this study, our objective was to investigate the relationship between having SAD and the use of emotion regulation strategies. We designed a Web Application featuring ten different multimedia examples of social situations. Individuals could rate the correspondence of their possible response to any of the suggested emotion regulation strategies, that is, suppression, avoidance, and reappraisal.

A significant difference in strategies among healthy and socially anxious individuals showed that individuals with SAD were more likely to use suppression and avoidance strategies. In contrast, individuals without SAD (healthy controls) were more likely to use adaptive reappraisal strategies. The effect sizes also showed significant differences between the two groups. Furthermore, the avoidance and suppression strategies are significantly correlated, but no correlation or negative correlation was observed between these two strategies and the reappraisal. The results are aligned with previous research and show a significant difference between healthy people and people with SAD (Anderson et al., 2008). Moreover, Cronbach’s alpha coefficient for each strategy was reliable.

The data also revealed a significant difference between men and women in their use of avoidance strategies. However, based on the results, we found no differences between the other two strategies for men and women. Future studies are necessary to assess possible differences. Furthermore, our age-limited population did not show significant differences within this age range, specifically between the ages of 18 and 35. Future studies could investigate differences between children, young adults and older ones, as well as scenarios that should be developed considering age-related social interactions.

In addition, we used machine learning algorithms to train a screening tool for individuals with SAD. We examined RF, SVM, KNN, and perceptron algorithms. All models were tuned using repeated 5-fold stratified cross-validation. The results showed that no single algorithm was uniformly superior across all metrics. Instead, each model emphasized different trade-offs: KNN achieved the highest F1 score, recall and accuracy, making it advantageous when minimizing missed cases; RF attained the highest precision and tied with SVM in the ROC AUC, highlighting its strength to reduce false positives and offering interpretability through feature importance. Finally, results shows that Perceptron performed consistently weaker across metrics. These results suggest that the choice of model in practice should be guided by clinical priority, whether it is avoiding false negatives, minimizing false positives, or maximizing discrimination ability, rather than by a single metric alone. Importantly, overall performance indicates that Multimedia based machine learning screening can reach a level of accuracy that can complement traditional diagnostic tools.

The advantage of the proposed web-based application is its wide accessibility and availability across different devices at any time and from any location. Individuals who may be screened for SAD should consult an expert for further evaluation and diagnosis. Thus, it handles the difficulty of accessing patients with SAD who mainly hesitate to communicate with people or experts with SAD (Chenjing, 2010). In the future, other emotion regulation strategies, such as attentional deployment and experiential avoidance, could be explored as potential tools for screening SAD. Additionally, a broader set of diagnostic criteria may be employed to enable more comprehensive screening, including assessments of individuals’ fear levels in social situations or the integration of artificial intelligence, such as facial emotion recognition during task participation. Another key consideration is the refinement or removal of scenarios that demonstrate problematic behavior or weak predictive performance.

This research was carried out in Iran’s society and among a Persian-speaking population. Given the importance of cultural factors in the use of emotion regulation strategies, it is recommended that future studies translate and apply this approach in the participants’ native language. Such an implementation should align with the target population’s culture and its results should be validated. The age group was also limited to young adults. Future studies may develop other versions of the web application, considering different age groups.

Taken together, our findings confirm that a lightweight, scenario-based web application combined with machine learning analytics can reliably distinguish individuals with and without SAD. A logical next step is to embed the same inference pipeline inside socially assistive robots (SAR), embodied agents that provide help through social rather than physical interactions (Feil-Seifer and Matarić, 2005). Presenting each scenario through speech, gaze, and expressive gestures would enable a robot to gather additional non-verbal cues, offer real-time coaching, and mitigate the risk of judgment based on face-to-face interactions. Early work shows that supportive SARs can reduce anxiety and increase self-disclosure in people with SAD (Rasouli et al., 2022). Therefore, future research should explore how robot appearance, movement, and adaptive feedback strategies can maximize engagement and accuracy, potentially paving the way for remote or in-clinic telepsychology services.

## Data Availability

The original contributions presented in the study are included in the article/supplementary material, further inquiries can be directed to the corresponding author.
